# Causal association between obesity and hypothyroidism: a two-sample bidirectional Mendelian randomization study

**DOI:** 10.3389/fendo.2023.1287463

**Published:** 2024-01-08

**Authors:** Yingkun Qiu, Qinyu Liu, Yinghua Luo, Jiadi Chen, Qingzhu Zheng, Yuping Xie, Yingping Cao

**Affiliations:** ^1^ Department of Clinical Laboratory, Fujian Medical University Union Hospital, Fuzhou, China; ^2^ Department of Endocrinology, Shengli Clinical Medical College of Fujian Medical University, Fuzhou, China

**Keywords:** Mendelian randomization, obesity, body mass index, waist circumference, hypothyroidism

## Abstract

**Introduction:**

Previous observational studies have reported a positive correlation between obesity and susceptibility to hypothyroidism; however, there is limited evidence from alternative methodologies to establish a causal link.

**Methods:**

We investigated the causal relationship between obesity and hypothyroidism using a two-sample bidirectional Mendelian randomization (MR) analysis. Single-nucleotide polymorphisms (SNPs) associated with obesity-related traits were extracted from a published genome-wide association study (GWAS) of European individuals. Summarized diagnostic data of hypothyroidism were obtained from the UK Biobank. Primary analyses were conducted using the inverse variance-weighted (IVW) method with a random-effects model as well as three complementary approaches. Sensitivity analyses were performed to ascertain the correlation between obesity and hypothyroidism.

**Results:**

MR analyses of the IVW method and the analyses of hypothyroidism/myxedema indicated that body mass index (BMI) and waist circumference (WC) were significantly associated with higher odds and risk of hypothyroidism. Reverse MR analysis demonstrated that a genetic predisposition to hypothyroidism was associated with an increased risk of elevated BMI and WC, which was not observed between WC adjusted for BMI (WCadjBMI) and hypothyroidism.

**Discussion:**

Our current study indicates that obesity is a risk factor for hypothyroidism, suggesting that individuals with higher BMI/WC have an increased risk of developing hypothyroidism and indicating the importance of weight loss in reducing the risk of hypothyroidism.

## 1 Introduction

Hypothyroidism is a condition characterized by the insufficient production of thyroid hormones, which can result in a variety of non-specific symptoms (1–4). Geographical variations in disease classification, poorly characterized and heterogeneous study cohorts, variability in the sensitivity of previous thyroid function assessment methods, and disparities in iodine intake are factors contributing to the disparities in hypothyroidism prevalence (5). A screening study in the United States showed that the prevalence of overt and subclinical hypothyroidism were 0.4% and 9%, respectively (5). A meta-analysis conducted in Europe and the National Health and Nutrition Examination Survey (NHANES III) in the US estimated similar prevalence rates, and it appears even higher in China (6–8). This prevalence has gradually increased over recent years with females and older adults being the primary affected populations (9).

The manifestations of hypothyroidism can vary in severity ranging from mild cases with minimal or almost no symptoms to severe cases that may include life-threatening myxedema (1, 10, 11). The etiology of hypothyroidism can be attributed to various factors including chronic autoimmune thyroiditis, iodine metabolism disorders, post-radiotherapy or post-thyroidectomy, genetic diseases, infections and/or inflammation, medication, and the presence of peripheral tissue-related consumptive and resistance-related hypothyroidism, which are crucial to consider.

Moreover, obesity represents a chronic inflammatory state that can be assessed using measurements such as body mass index (BMI), waist circumference (WC) and WC adjusted for BMI (WCadjBMI) in clinical practice as indicators for evaluating and managing obesity. Recently, the association between obesity and hypothyroidism has received considerable attention (12–14); however, the underlying causes and effects of obesity and hypothyroidism remain unclear. Some studies have suggested that patients with hypothyroidism typically experience weight gain, which is difficult to control (3, 15). Mehran et al. demonstrated that overt and subclinical hypothyroidism, especially in the elderly, may be associated with metabolic syndromes (16). A meta-analysis demonstrated that subclinical hypothyroidism is significantly associated with an elevated risk of obesity, hypertension, elevated triglyceride levels, and reduced high-density lipoprotein cholesterol levels (17); however, other studies have indicated that obesity increases the risk of hypothyroidism (18–20). In contrast, another study reported an inverse association between a high BMI and the prevalence of subclinical hypothyroidism in males (21).

Inferences made from observational studies regarding causality are constrained and not entirely trustworthy, possibly due to unknown or unmeasured confounding factors that may have influenced the results. Therefore, Mendelian randomization (MR) analysis was used to integrate genomic data into conventional epidemiological studies to facilitate the inference of causal effects between obesity and hypothyroidism (22, 23). By leveraging genetic variants as instrumental variables (IVs), this approach provides a framework for inferring causal relationships in observational data, mitigating potential biases, and enhancing the credibility of the findings (24, 25). Using MR analysis, we investigated the causal relationship between obesity and hypothyroidism, which allowed us to ascertain the directionality of the association. This approach will be valuable for developing treatment and care strategies for patients with hypothyroidism.

## 2 Materials and methods

### 2.1 Study design

**Figure 1:**
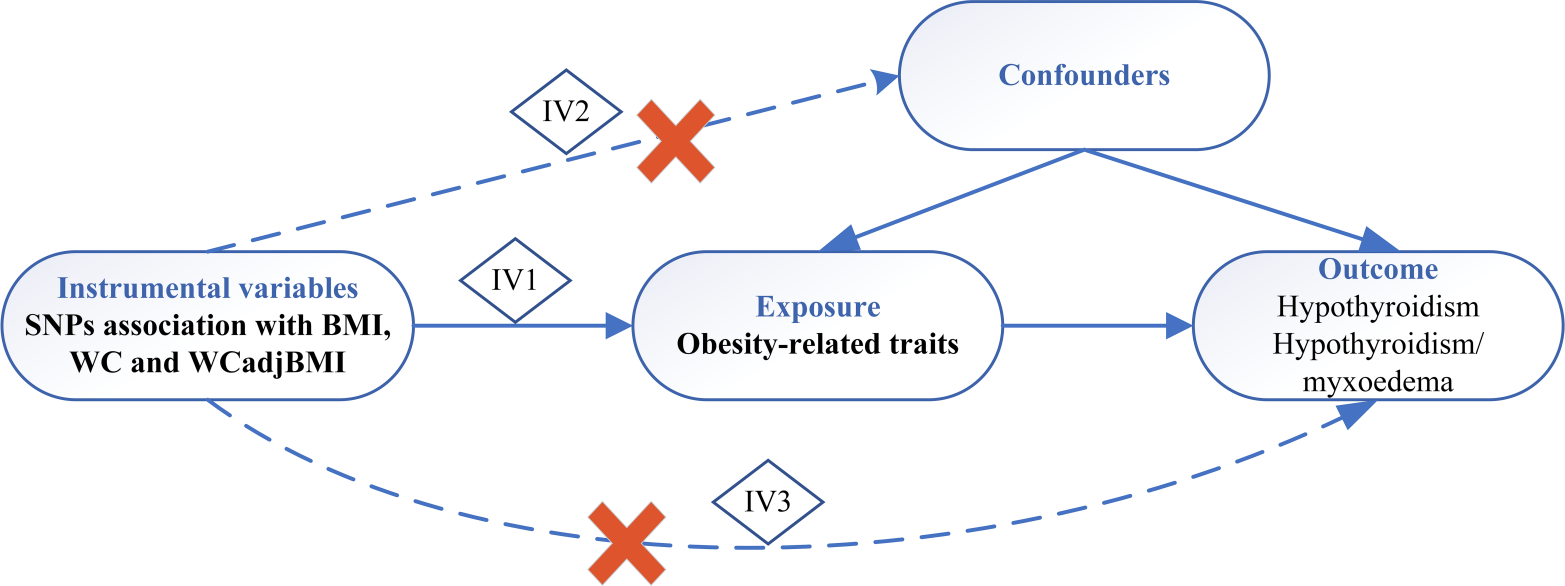
Schematic diagram of Mendelian randomization study. IV, instrumental variable; IV 1-3 represents 3 core assumptions, SNP, single nucleotide polymorphism; BMI, body mass index; WC, waist circumference; WCadjBMI, waist circumference adjusted for body mass index.

We conducted a two-sample MR study to assess the causal effect of obesity-related parameters on the development of hypothyroidism (**Figure 1**). The MR analysis relies on three core assumptions:

1. Genetic variants are associated with exposure, and there should be a strong and robust association between the genetic variants used as IVs and the exposure of interest (e.g., obesity-related parameters).
2. Genetic variants are independent of confounders, and the genetic variants used in the MR analysis should be independent of factors that could confound the association between exposure and outcome (e.g., other risk factors or comorbidities).
3. Genetic variants influence the outcome only through exposure and should have a causal effect on the outcome (e.g., hypothyroidism) solely through their influence on exposure (e.g., obesity-related parameters) without any alternative pathways or reverse causation.

By satisfying these assumptions, MR analysis can provide insight into the causal relationship between exposure and the outcome of interest (26). Data from published research and publicly available genome-wide association studies (GWAS) were used for the subsequent analyses.

### 2.2 Data source

The exposure variable data for the genetic variants associated with BMI, WC, and WCadjBMI were obtained from a GWAS meta-analysis conducted by the Genetic Investigation of Anthropometric Traits consortium (27, 28). This consortium combines and analyzes data from multiple studies to identify genetic variants associated with anthropometric traits including obesity. Outcome variable data were obtained from the UK Biobank, a large-scale cohort study that enrolled more than 500,000 men and women from the general population of the United Kingdom between 2006 and 2010. Characteristics of the data sources are summarized in **Table 1**. Further details regarding these data sources can be found in the original publications associated with each study. No further ethics approval or informed consent was necessary as this study relied on publicly available databases.

**Table 1:** Characteristics of studies used for primary MR analysis.

GWAS ID	Traits	Reference	Consortium	Sample size	Ncase	Ncontrol	Population
ieu-b-40	BMI	Yengo, L et al., 2015	GIANT	339,224	NA	NA	European
Ieu-a-61	WC	Shungin D et al., 2015 (28)	GIANT	232,101	NA	NA	European
ieu-a-67	WCadjBMI	Shungin D et al., 2015 (28)	GIANT	231,353	NA	NA	European
ukb-b-4226	Hypothyroidism	Ben Elsworth et al., 2018	MRC-IEU	463,010	9,674	453,336	European
ukb-a-77	Hypothyroidism/myxedema	Neale et al., 2019	Neale Lab	337,159	16,376	320,783	European

GWAS, Genome wide association study; BMI, Body mass index; WC, Waist circumference; WCadjBMI, Waist circumference adjusted for BMI; GIANT, Genetic Investigation of Anthropometric Traits; ukb, UK biobank; NA, not available; MRC-IEU, MRC Integrative Epidemiology Unit.

### 2.3 IV selection

Several high-quality procedures were used to screen the best IVs to ensure the integrity and precision of the findings. The procedures included the following criteria:

1. Predictiveness of exposure: Only single nucleotide polymorphisms (SNPs) that were significantly associated with the exposure variables (i.e., BMI, WC, and WCadjBMI) at a genome-wide significance level (*P <* 5 × 10E−8) were considered.
2. Independence from confounders: Linkage disequilibrium (LD) clumping was performed to ensure independence from potential confounding factors. SNPs were examined for their independence by setting a threshold of LD R^2^ < 0.001 and an LD distance > 1000 kb using the “clump” function.
3. Absence of alternative pathways: The selected SNPs did not have any independent pathways that could influence the outcome variable (i.e., hypothyroidism) apart from the exposure variable.

Furthermore, the F-statistics of the SNPs were calculated to assess instrument strength. SNPs with an F-statistic < 10, indicating a weak correlation, were considered and subsequently removed from the analysis.

These rigorous procedures were implemented to select IVs that met the criteria of predictiveness, independence, and absence of alternative pathways, ultimately ensuring the robustness of the IV analysis. Specific details regarding the LD clumping, R^2^ extraction, and F-statistic calculations can be found in the original study (29).

### 2.4 Statistical analysis

The MR analysis was performed using the “TwoSampleMR” package in R version 4.3.0 (R Foundation for Statistical Computing, Vienna, Austria). The primary analysis employed the inverse variance-weighted (IVW) method to assess the causal relationship between obesity and hypothyroidism. The IVW method calculates the exposure-outcome effect for each SNP using the Wald ratio method and conducts a weighted linear regression with a forced intercept of zero. This method is known for its higher estimation accuracy and test power when the IVs satisfy three assumptions (30). To account for potential interference from unknown and unmeasurable confounders, MR-Egger regression (MR-Egger) was performed (31). Additionally, we used the weighted median method, which provides consistent estimates of causality even when up to 50% of the information comes from invalid IVs (32). Visualizations such as funnel, scatter, and forest plots were used to assess the heterogeneity in the causal estimates derived from different genetic instruments. The weighted mode method was also applied (33). To evaluate heterogeneity, the modified Cochran’s Q statistic and leave-one-SNP-out analyses were employed. If the p-values were higher than 0.05, with no evidence of heterogeneity, the fixed-effects IVW approach was considered the main approach. If substantial heterogeneity was present (i.e., *P <* 0.05), the random effects IVW approach was used.

## 3 Results

### 3.1 Validity of IVs

SNPs representing variations in BMI, WC, and WCadjBMI were selected as IVs for the analysis of single-trait hypothyroidism and combined trait hypothyroidism/myxedema. Detailed information regarding these SNPs, including SNP identifiers, beta coefficients, standard errors (SE), and p-values, is provided in **Supplementary Tables 1, 2**. The selected SNPs were confirmed to be independent (not in LD). Moreover, all included IVs demonstrated F-statistics > 10, indicating the absence of weak IV bias in the results and ensuring the reliability of our findings.

### 3.2 Causal relationship between obesity and hypothyroidism

#### 3.2.1 BMI on hypothyroidism

**Figure 2:**
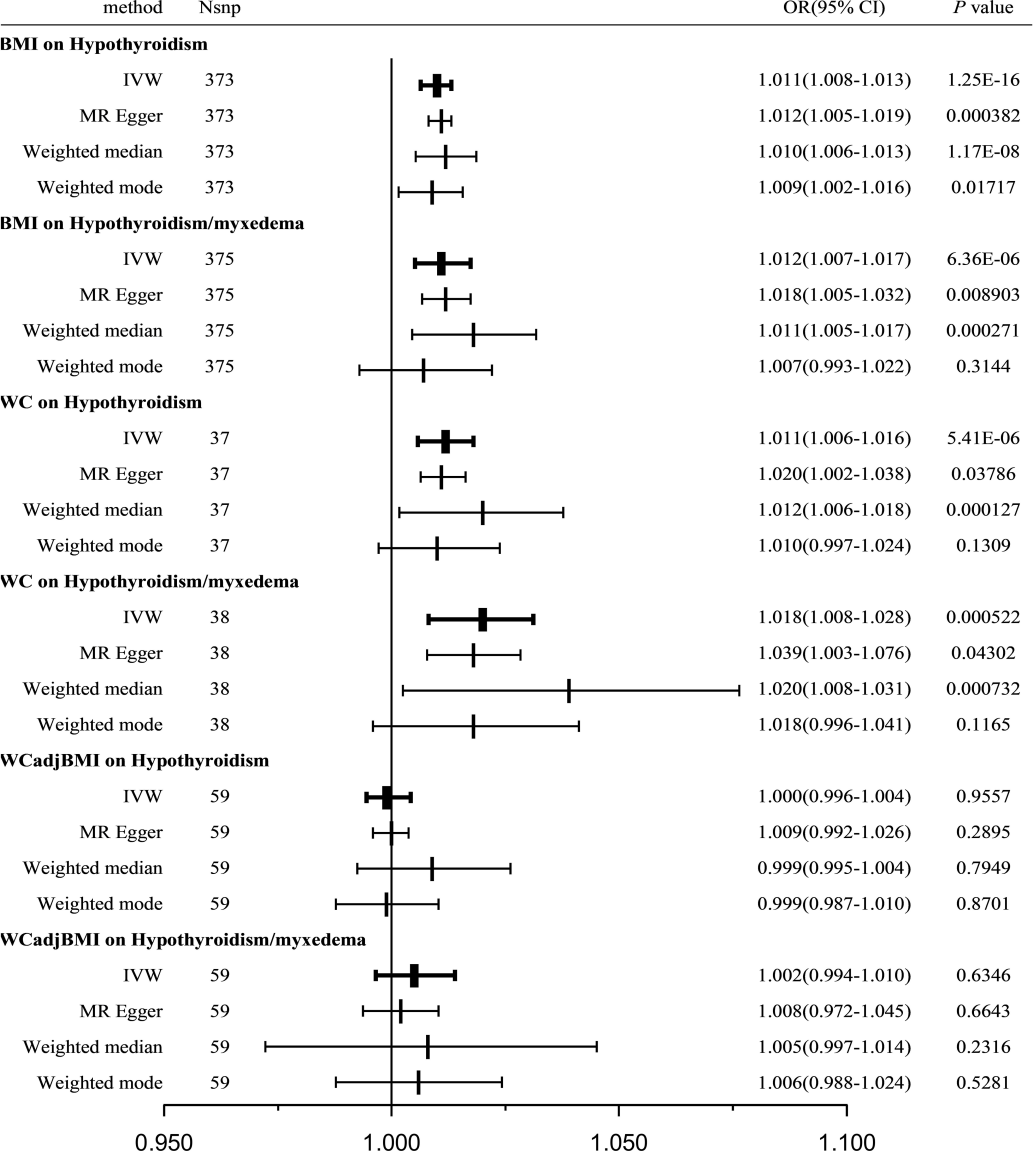
Causal estimates given as OR and 95%CI for the effect of obesity-related traits on hypothyroidism and hypothyroidism/myxedema. BMI, body mass index; WC, waist circumference; WCadjBMI, waist circumference adjusted for body mass index; IVW, Inverse variance weighted; OR, odds ratio; 95%CI, 95% confidence intervals.

In total, 373 SNPs associated with BMI were identified in relation to hypothyroidism. Cochran’s Q statistics revealed significant heterogeneity among the included SNPs (Q = 592.9039, *P <* 0.001). The IVW method with a multiplicative random-effects model was used and we found a significant causal effect between BMI and hypothyroidism (odds ratio [OR] = 1.011; 95% CI = 1.008-1.013; *P =* 1.25E-16). The sensitivity analysis demonstrated no substantial horizontal pleiotropy (regression intercept of the MR-Egger test was close to zero, *P =* 0.6716; **Table 2**). Consistent results were observed using the weighted median (OR = 1.010; 95% CI = 1.006-1.013; *P =* 1.17E-08) and MR-Egger (OR = 1.012; 95% CI = 1.005-1.019; *P =* 3.8E-4) methods. The weighted mode estimates also exhibited a significant causal effect in the same direction (OR = 1.009; 95% CI = 1.002-1.016; *P <* 0.05; **Figure 2**).

**Table 2:** Heterogeneity and pleiotropy analysis of obesity with hypothyroidism using different analytical methods.

Exposure traits	Outcome traits	MR methods	Cochran Q statistic	Heterogeneity p-value	Directional horizontal pleiotropy p-value
BMI	hypothyroidism	IVW	592.9039	<0.001	0.6716
	hypothyroidism/myxededma	IVW	842.0871	<0.001	0.3442
WC	hypothyroidism	IVW	56.2699	0.0169	0.3552
	hypothyroidism/myxededma	IVW	82.2342	<0.001	0.2545
WCadjBMI	hypothyroidism	IVW	85.5966	0.0107	0.2705
	hypothyroidism/myxededma	IVW	127.5437	<0.001	0.7382

BMI, Body mass index; WC, Waist circumference; WCadjBMI, Waist circumference adjusted for BMI; MR, Mendelian Randomization; IVW, Inverse variance weighted.

Furthermore, a verification test was conducted using another outcome dataset, hypothyroidism/myxedema, with 375 SNPs associated with BMI. Similar to the previous analysis, Cochran’s Q statistics indicated significant heterogeneity among the included SNPs (Q = 842.0871, *P <* 0.001). The IVW method with a multiplicative random-effects model revealed a significant causal effect between BMI and hypothyroidism/myxedema (OR = 1.012; 95% CI = 1.007-1.017; *P =* 6.36E-06). Sensitivity analysis showed no substantial horizontal pleiotropy (regression intercept of the MR-Egger test was close to zero, *P =* 0.3442; **Table 2**). Consistent results were observed using the weighted median (OR = 1.011; 95% CI = 1.005-1.017; *P =* 2.7E-04) and MR-Egger (OR = 1.018; 95% CI = 1.005-1.032; *P =* 8.9E-3) methods. The weighted mode estimates exhibited a similar trend but lacked statistical significance (OR = 1.007; 95% CI = 0.993-1.022; *P =* 0.3144; **Figure 2**).

**Supplementary Figures 1**, **2** present forest, scatter, and funnel plots as well as the leave-one-out analysis. The overall findings from the MR analysis support a positive causal effect between BMI and the risk of hypothyroidism.

#### 3.2.2 WC on hypothyroidism

A total of 37 WC-related SNPs were examined for their association with hypothyroidism. Significant heterogeneity was observed among the SNPs (Q = 56.2699, *P =* 0.0169). Using the IVW method with a random-effects model, a significant and positive causal effect was found between WC and hypothyroidism (OR = 1.011; 95% CI = 1.006-1.016; *P =* 5.41E-06). Sensitivity analysis revealed no remarkable horizontal pleiotropy (MR-Egger intercept close to zero, *P =* 0.3552; **Table 2**). Consistent results were obtained using the weighted median (OR = 1.012; 95% CI = 1.006-1.018; *P =* 1.27E-04) and MR-Egger (OR = 1.020; 95% CI = 1.002-1.038; *P =* 0.03786) methods; however, the weighted-mode estimates did not show statistical significance (OR = 1.010; 95% CI = 0.997-1.024; *P =* 0.1309; **Figure 2**).

A verification test was conducted using another outcome dataset, hypothyroidism/myxedema, with 38 WC-related SNPs. Similar to the previous analysis, significant heterogeneity was observed among the included SNPs (Q = 82.2342, *P =* 2.77E-05). IVW analysis using a random-effects model revealed a significant causal effect between WC and hypothyroidism/myxedema (OR = 1.018; 95% CI = 1.008-1.028; *P =* 5.22E-04). Sensitivity analysis indicated no remarkable horizontal pleiotropy (MR-Egger intercept close to zero, *P =* 0.2545; **Table 2**). The weighted median (OR = 1.020; 95% CI = 1.008-1.031; *P =* 7.32E-04) and MR-Egger (OR = 1.039; 95% CI = 1.003-1.076; *P =* 0.04302) methods consistently supported this causal effect; however, the weighted-mode estimates did not show statistical significance (OR = 1.008; 95% CI = 0.996-1.041; *P =* 0.1165; **Figure 2**).

The forest, scatter, and funnel plots, and the leave-one-out analysis, supporting a positive causal effect between WC and the risk of hypothyroidism are presented in **Supplementary Figures 3**, **4**.

#### 3.2.3 WCadjBMI on hypothyroidism

A total of 59 WCadjBMI-related SNPs were examined for their association with hypothyroidism. Significant heterogeneity was observed among SNPs (Q = 85.5966, *P =* 0.0107); however, IVW analysis using a random-effects model indicated no significant association between WCadjBMI and hypothyroidism (OR = 1.002; 95% CI = 0.994-1.010; *P =* 0.6346). Sensitivity analysis showed no remarkable horizontal pleiotropy (MR-Egger intercept close to zero, *P =* 0.2705; **Table 2**). Consistent results were obtained using weighted median, MR-Egger, and weighted mode estimates, all of which supported a null association.

A verification test was conducted using another outcome dataset, hypothyroidism/myxedema, with 59 WCadjBMI-related SNPs. Similar to the previous analysis, significant heterogeneity was observed among the included SNPs (Q = 127.5437, *P =* 4E-07). The IVW analysis indicated a null association between WCadjBMI and hypothyroidism (OR = 1.018; 95% CI = 1.008-1.028; *P =* 5.22E-04). Sensitivity analysis showed no remarkable horizontal pleiotropy (MR-Egger intercept close to zero, *P =* 0.2545; **Table 2**). This null association was consistent with the weighted median, MR-Egger test, and weighted mode estimates (**Figure 2**).

The forest, scatter, and funnel plots, and leave-one-out analysis, further supporting the lack of a causal relationship between WCadjBMI and hypothyroidism are shown in **Supplementary Figures 5**, **6**.

#### 3.2.4 Hypothyroidism on obesity

A total of 24, 26, and 26 SNPs related to hypothyroidism were examined for their association with BMI, WC, and WCadjBMI, respectively. Significant heterogeneity was observed among the included SNPs, except for WC as an exposure variable (**Table 3**).

**Table 3:** Heterogeneity and pleiotropy analysis of hypothyroidism with obesity using different analytical methods.

Exposure traits	Outcome traits	MR methods	Cochran Q statistic	Heterogeneity p-value	Directional horizontal pleiotropy p-value
hypothyroidism	BMI	IVW	75.1653	<0.001	0.6112
	WC	IVW	32.3268	0.1488	0.3659
	WCadjBMI	IVW	41.0647	0.0226	0.2161
hypothyroidism/myxededma	BMI	IVW	215.9188	<0.001	0.3627
	WC	IVW	75.5121	0.0509	0.0344
	WCadjBMI	IVW	98.3950	<0.001	0.3410

BMI, Body mass index; WC, Waist circumference; WCadjBMI, Waist circumference adjusted for BMI; MR, Mendelian Randomization; IVW, Inverse variance weighted.

**Figure 3:**
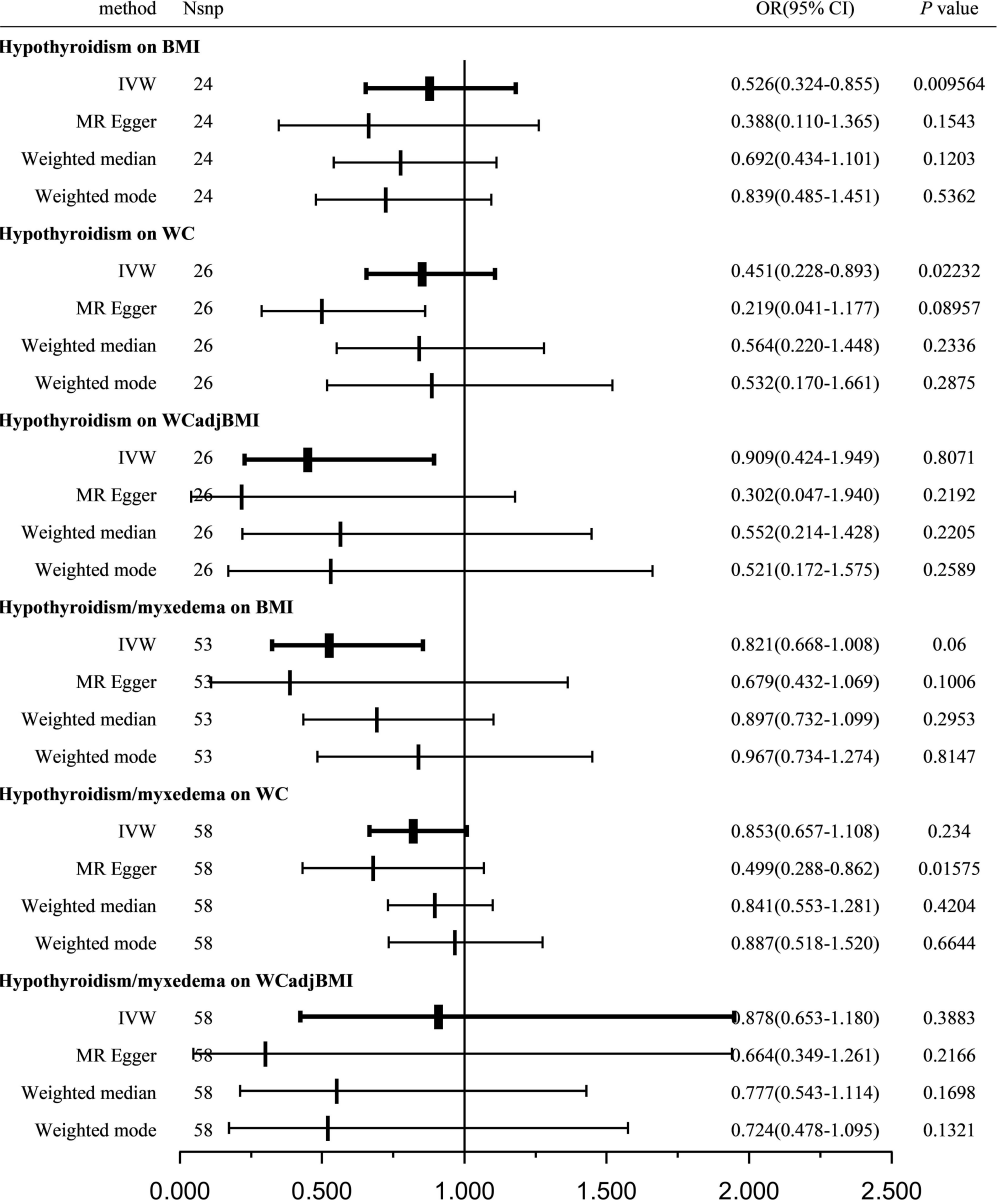
Causal estimates given as OR and 95%CI for the effect of hypothyroidism and hypothyroidism/myxedema on obesity-related traits. BMI, body mass index; WC, waist circumference; WCadjBMI, waist circumference adjusted for body mass index; IVW, Inverse variance weighted; OR, odds ratio; 95%CI, 95% confidence intervals.

Using the IVW method with a random-effects model, the analysis showed a significant causal effect between hypothyroidism and BMI (OR = 0.526; 95% CI = 0.324-0.855; *P =* 0.009564) and WC (OR = 0.451; 95% CI = 0.228-0.893; *P =* 0.02232); however, other MR analysis methods did not reveal a significant correlation between the exposure variables and outcomes. Furthermore, our analysis did not reveal any significant genetic correlation between hypothyroidism and WCadjBMI, indicating that there is no substantial shared genetic influence between these two factors (OR = 0.909; 95% CI = 0.424-1.949; *P =* 0.8071; **Figure 3**).

Sensitivity analysis indicated no remarkable horizontal pleiotropy for any of the three exposure variables (*P*_BMI_ = 0.6112, *P*_WC_ = 0.3659, and *P*_WCadjBMI_ = 0.2161; **Table 3**).

The forest, scatter, and funnel plots, and leave-one-out analysis, which further support the lack of a strong positive link between hypothyroidism and obesity are demonstrated in **Supplementary Figures 7**–**9**.

#### 3.2.5 Hypothyroidism/myxedema on obesity

A total of 53, 58, and 58 SNPs related to hypothyroidism/myxedema were examined for their association with BMI, WC, and WCadjBMI, respectively. Significant heterogeneity was observed among the included SNPs, except for WC as an exposure variable (**Table 3**).

Using the IVW method with a random-effects model, the analysis indicated that hypothyroidism/myxedema was not significantly associated with BMI (OR = 0.821; 95% CI = 0.668-1.008; *P =* 0.06), WC (OR = 0.853; 95% CI = 0.657-1.108; *P =* 0.234), or WCadjBMI (OR = 0.878; 95% CI = 0.653-1.180; *P =* 0.3883; **Figure 3**).

Sensitivity analysis showed no remarkable horizontal pleiotropy except for WC as an exposure variable (*P*_BMI_ = 0.3627, *P*_WC_ = 0.0344, and *P*_WCadjBMI_ = 0.341; **Table 3**).

The forest, scatter, and funnel plots, and leave-one-out analysis, which further support the finding that no significant causal effect was found between hypothyroidism/myxedema and any of the obesity traits tested, are presented in **Supplementary Figures 10**–**12**.

**Figure 4:**
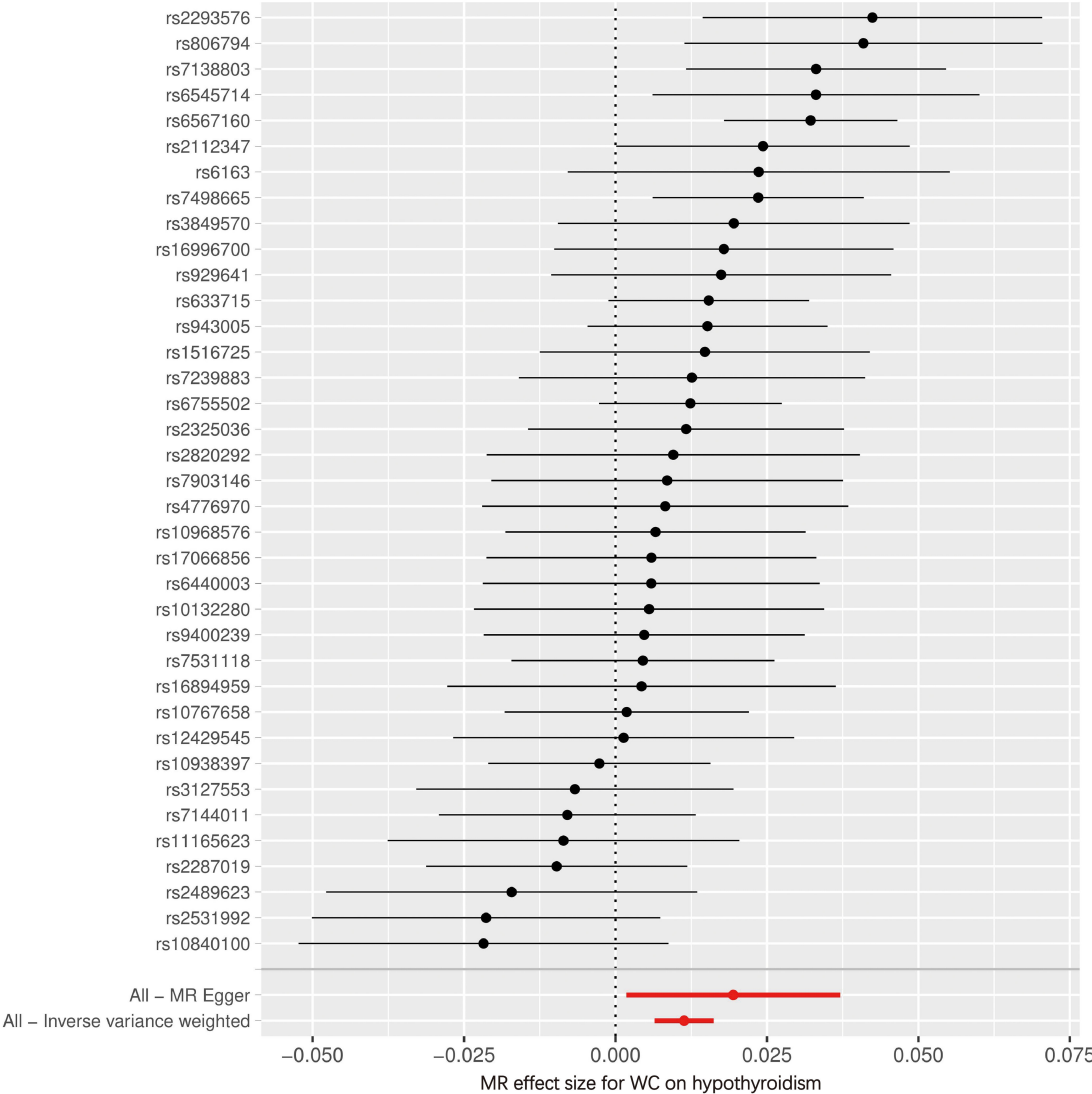
Forest plot of SNPs associated with WC and the risk of hypothyroidism. WC, waist circumference; MR, Mendelian randomization.

**Figure 5:**
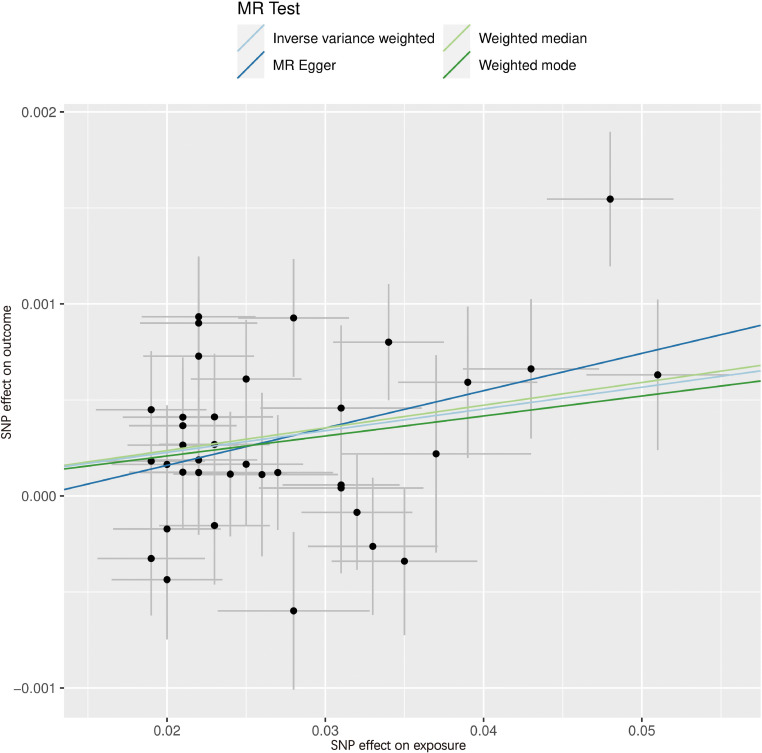
Scatter plot of SNPs associated with WC and the risk of hypothyroidism. The slope of each line corresponds to the causal estimate using different MR methods. SNP, single nucleotide polymorphism; MR, Mendelian randomization.

**Figures 4**, **5** serve as illustrative examples that showcase the relationship between exposure-related SNPs and the outcome. To visually represent the causal estimates and their corresponding confidence intervals for each IV, we employ a forest plot (**Figure 4**). This aids in assessing both individual and overall effects within the analysis. Additionally, a scatter plot (**Figure 5**) visually displays the associations between the IVs and the exposure and outcome variables, facilitating an examination of the strength, direction, and potential causal effects of these relationships. The lines of varying colors in the plot represent the regression slopes fitted by different MR methods.

## 4 Discussion

In our current MR study, we analyzed the SNPs associated with BMI, WC, and WCadjBMI, which represent overall and central obesity. The nature of random genotype distribution in the general population and fixed germline genotypes makes MR analysis less prone to confounding and reverse causation than observational studies. We investigated and found significant and positive causal effects among BMI, WC, and the risk of hypothyroidism (Unspecified Hypothyroidism ICD 10 code E03.9); Although reverse MR analyses revealed a reverse causation effect between one hypothyroidism trait and both BMI and WC, this effect was observed only when employing the IVW method. However, when considering the consistent findings from Forward MR analyses, the results obtained from reverse MR analyses for both hypothyroidism traits, and acknowledging the limitations of the IVW method, we can conclude that obesity has a causal effect on hypothyroidism. Our findings are consistent with those of previous observational studies (12, 34, 35). Furthermore, when we specifically examined WCadjBMI as the exposure variable, we did not observe any significant association, indicating that central obesity may not be a risk factor for hypothyroidism. Importantly, using genetic instruments for the WCadjBMI may introduce bias in MR estimates because of their association with a lower BMI (36).

Primary overt hypothyroidism is characterized by the insufficient production of T3 and T4 hormones and increased levels of thyroid-stimulating hormone (TSH) in the bloodstream. Previous studies have established a significant bidirectional relationship between obesity and hypothyroidism (37–39). Weight gain is a common symptom of hypothyroidism and is more prevalent in obese individuals; however, differentiating weight gain from that caused by low thyroxine levels or excess energy remains challenging. Therefore, it is crucial to determine whether there is a causal connection between obesity and hypothyroidism as well as the underlying causes and effects. Large-scale studies have indicated a potential causal relationship between genetically predicted high BMI and WC and the risk of hypothyroidism; however, this association became insignificant when WC was adjusted for BMI, suggesting that the causal effect of central obesity might primarily depend on overall obesity. Despite several observational findings suggesting a positive association between obesity and hypothyroidism, the exact causality remains unclear. Several studies have demonstrated a 70% increased risk of subclinical hypothyroidism in obese individuals (37, 40). In obese children and adolescents, approximately 32% of patients exhibit subclinical hypothyroidism along with higher body fat levels. Subclinical hypothyroidism is significantly associated with weight and risk factors for being overweight and obese. Overweight euthyroid patients also showed a correlation between elevated TSH levels and fat accumulation, with TSH levels positively associated with weight gain (18–20). Various studies have shown that bariatric surgery in obese patients improves their thyroid profile (41–43). A meta-analysis revealed that bariatric surgery has a positive impact on subclinical hypothyroidism, reducing TSH levels and lowering the required dose of thyroid hormone replacement therapy in patients with severe obesity (44). Another meta-analysis involving 22 studies on overweight children and adults reported a relative risk of 3.1 for hypothyroidism and 1.7 for subclinical hypothyroidism (38). Further research is needed to determine the obesity traits or fat distribution patterns that better explain the increased risk of hypothyroidism.

Although the precise underlying mechanisms by which obesity affects hypothyroidism remain unclear, previous studies have suggested that obesity contributes to this condition through various pathways, such as the adipokine pathway, chronic low-grade inflammation, and autoimmune dysfunction. Leptin resistance is common in obese individuals and shows a positive correlation with BMI (45) Increased leptin levels stimulate the secretion of thyroid-stimulating hormone-releasing hormone (TRH) and TSH by interacting with specific leptin receptors in the hypothalamic arcuate nucleus, paraventricular nucleus, dorsomedial nucleus, and lateral nucleus of the hypothalamus (46–49). Leptin signaling primarily utilizes the JAK/STAT pathway to regulate TRH expression in the hypothalamic paraventricular nucleus (50). Furthermore, leptin plays a crucial role in the development of low-grade systemic inflammation in obesity. Knocking out leptin receptors in leukocytes of DIO mice significantly reduces inflammation in white adipose tissue (51). In addition to leptin, adipose tissue also secretes over 600 different bioactive molecules, including cytokines and chemokines (52–54). These adipokines, such as TNF-α, IL-1, and IL-6, hinder the expression of sodium iodine transporter mRNA and iodine uptake in Fisher rat thyroid cell lines and human thyroid cells. Consequently, this leads to a diminished iodine uptake capacity in both human and rat thyroid cells (55, 56). Due to the primary role of leptin in this process, the systemic inflammatory state associated with overweightness suggests an elevated risk of autoimmune thyroid diseases, including Hashimoto’s thyroiditis, which is the primary cause of hypothyroidism (46, 57–59). On the contrary, adiponectin, an anti-inflammatory cytokine, regulates metabolic processes and the adiponectin-leptin ratio serves as a marker of obesity-related inflammation (60, 61). In summary, all above highlight the close relationship between obesity and hypothyroidism.

Unlike previous observational studies that are susceptible to various confounding factors such as environmental influences, immune responses, and genetic variations, conducting large-scale RCTs in clinical practice is challenging. Our present study effectively addressed the limitations of observational studies by minimizing potential confounding factors and reverse causality. By utilizing publicly available GWAS data, we benefitted from a large sample size and focused on a European population, thereby reducing the impact of biased results owing to the ethnic factors. Sensitivity analyses were performed to ensure stability of the findings. Heterogeneity tests, such as the Q-*p* values of IVW and MR-Egger, yielded values of less than 0.05. Additionally, the results of the random-effects model reinforced the association between increased BMI and WC and an elevated risk of hypothyroidism. We also assessed pleiotropy and demonstrated no evidence of pleiotropic effects. Furthermore, the sensitivity analysis using the “leave-one-out” method confirmed the robustness and reliability of the findings. MR research offers advantages in studying etiology in epidemiology as it is not constrained by ethical considerations and financial limitations. We utilized two independent hypothyroidism datasets obtained from different authors and consortiums as outcome variables. Moreover, our analysis yielded compelling evidence supporting BMI and WC as significant risk factors for hypothyroidism.

Importantly, our current study also has certain limitations. The study only included European populations for both the exposure and outcome assessment, which limits our ability to generalize the findings to non-European populations. Next, the hypothyroidism dataset we utilized was based on clinical coding diagnoses, which, although relatively accurate for establishing the causal relationship of hypothyroidism, may have overlooked subclinical hypothyroidism patients diagnosed based on TSH/TFTs (thyroid-stimulating hormone/thyroid function tests) levels. Notably, elevated concentrations of TSH in obese individuals do not consistently indicate the presence of hypothyroidism, as peripheral thyroid hormones (T4 and T3) can exhibit increased, decreased, or normal levels (62–64). The specific impact of central obesity or peripheral obesity on the risk of hypothyroidism has not been further investigated. Lastly, the extracted GWAS data did not incorporate a subgroup analysis considering factors such as the subjects’ sex, age, medical history, degree of obesity (stratified by BMI or WC), and other relevant variables. IVs obtained from more refined hierarchical data can improve the reliability of the results, which is the direction of follow-up research.

In summary, we used a two-sample MR approach to assess the causal relationship between obesity and hypothyroidism (including hypothyroidism and myxedema). Obesity-related genetic data from three GWAS datasets were used as exposure variables, and the analysis was conducted using the IVW and MR-Egger methods. Sensitivity analyses were performed to examine pleiotropy, and no evidence of pleiotropic effects was found. The random-effects model confirmed that obesity was a risk factor for hypothyroidism. Therefore, weight loss may be beneficial in reducing the risk of hypothyroidism, and in clinical practice, weight management in patients undergoing treatment for hypothyroidism is recommended. Further studies are required to investigate the mechanisms underlying this relationship.

## 5 Conclusions

In conclusion, this two-sample MR study supports a potential causal relationship between a genetic predisposition to obesity and the development of hypothyroidism. These findings highlight the importance of effective management strategies to reduce the risk of hypothyroidism in the individuals with obesity.

## Data availability statement

The original contributions presented in the study are included in the article/**Supplementary Material**. Further inquiries can be directed to the corresponding author.

## Ethics statement

Ethical approval was not required for the study involving humans in accordance with the local legislation and institutional requirements. Written informed consent to participate in this study was not required from the participants or the participants’ legal guardians/next of kin in accordance with the national legislation and the institutional requirements.

## Author contributions

YQ: Conceptualization, Data curation, Formal Analysis, Funding acquisition, Project administration, Software, Validation, Writing – original draft, Writing – review & editing. QL: Conceptualization, Data curation, Investigation, Methodology, Project administration, Validation, Visualization, Writing – review & editing, Writing – original draft. YL: Methodology, Software, Validation, Writing – review & editing. JC: Formal Analysis, Investigation, Writing – review & editing. QZ: Resources, Visualization, Writing – review & editing. YX: Data curation, Resources, Writing – review & editing. YC: Project administration, Supervision, Writing – review & editing.

## Supplementary material

Supplementary Figure 1 Complementary MR analyses of BMI with hypothyroidism. **(A)** Forest plot, it provides a visual representation of the causal estimates and their corresponding confidence intervals for each IV, aiding in the assessment of individual and overall effects in the analysis; **(B)** Scatter plot, it visually displays the associations between IVs and the exposure and outcome variables, facilitating the examination of the strength and direction of these relationships and aiding in the identification of potential causal effects. The regression slops fitted by different MR methods are represented by different MR methods are represented by lines of varying colors; **(C)** Funnel plot, it is used to visually assess the presence of publication bias and asymmetry in the distribution of estimated causal effects, helping to evaluate the reliability and potential bias in the analysis; **(D)** Leave-one-out analysis, it serves as a sensitivity analysis to assess the robustness and reliability of the causal estimates by systematically excluding individual instrumental variables and evaluating their impact on the overall findings.

Supplementary Figure 2 Complementary MR analyses of BMI with hypothyroidism/myxedema. **(A)** Forest plot, it provides a visual representation of the causal estimates and their corresponding confidence intervals for each IV, aiding in the assessment of individual and overall effects in the analysis; **(B)** Scatter plot, it visually displays the associations between IVs and the exposure and outcome variables, facilitating the examination of the strength and direction of these relationships and aiding in the identification of potential causal effects. The regression slops fitted by different MR methods are represented by different MR methods are represented by lines of varying colors; **(C)** Funnel plot, it is used to visually assess the presence of publication bias and asymmetry in the distribution of estimated causal effects, helping to evaluate the reliability and potential bias in the analysis; **(D)** Leave-one-out analysis, it serves as a sensitivity analysis to assess the robustness and reliability of the causal estimates by systematically excluding individual instrumental variables and evaluating their impact on the overall findings.

Supplementary Figure 3 Complementary MR analyses of WC with hypothyroidism. **(A)** Forest plot, it provides a visual representation of the causal estimates and their corresponding confidence intervals for each IV, aiding in the assessment of individual and overall effects in the analysis; **(B)** Scatter plot, it visually displays the associations between IVs and the exposure and outcome variables, facilitating the examination of the strength and direction of these relationships and aiding in the identification of potential causal effects. The regression slops fitted by different MR methods are represented by different MR methods are represented by lines of varying colors; **(C)** Funnel plot, it is used to visually assess the presence of publication bias and asymmetry in the distribution of estimated causal effects, helping to evaluate the reliability and potential bias in the analysis; **(D)** Leave-one-out analysis, it serves as a sensitivity analysis to assess the robustness and reliability of the causal estimates by systematically excluding individual instrumental variables and evaluating their impact on the overall findings.

Supplementary Figure 4 Complementary MR analyses of WC with hypothyroidism/myxedema. **(A)** Forest plot, it provides a visual representation of the causal estimates and their corresponding confidence intervals for each IV, aiding in the assessment of individual and overall effects in the analysis; **(B)** Scatter plot, it visually displays the associations between IVs and the exposure and outcome variables, facilitating the examination of the strength and direction of these relationships and aiding in the identification of potential causal effects. The regression slops fitted by different MR methods are represented by different MR methods are represented by lines of varying colors; **(C)** Funnel plot, it is used to visually assess the presence of publication bias and asymmetry in the distribution of estimated causal effects, helping to evaluate the reliability and potential bias in the analysis; **(D)** Leave-one-out analysis, it serves as a sensitivity analysis to assess the robustness and reliability of the causal estimates by systematically excluding individual instrumental variables and evaluating their impact on the overall findings.

Supplementary Figure 5 Complementary MR analyses of WCadjBMI with hypothyroidism. **(A)** Forest plot, it provides a visual representation of the causal estimates and their corresponding confidence intervals for each IV, aiding in the assessment of individual and overall effects in the analysis; **(B)** Scatter plot, it visually displays the associations between IVs and the exposure and outcome variables, facilitating the examination of the strength and direction of these relationships and aiding in the identification of potential causal effects. The regression slops fitted by different MR methods are represented by different MR methods are represented by lines of varying colors; **(C)** Funnel plot, it is used to visually assess the presence of publication bias and asymmetry in the distribution of estimated causal effects, helping to evaluate the reliability and potential bias in the analysis; **(D)** Leave-one-out analysis, it serves as a sensitivity analysis to assess the robustness and reliability of the causal estimates by systematically excluding individual instrumental variables and evaluating their impact on the overall findings.

Supplementary Figure 6 Complementary MR analyses of WCadjBMI with hypothyroidism/myxedema. **(A)** Forest plot, it provides a visual representation of the causal estimates and their corresponding confidence intervals for each IV, aiding in the assessment of individual and overall effects in the analysis; **(B)** Scatter plot, it visually displays the associations between IVs and the exposure and outcome variables, facilitating the examination of the strength and direction of these relationships and aiding in the identification of potential causal effects. The regression slops fitted by different MR methods are represented by different MR methods are represented by lines of varying colors; **(C)** Funnel plot, it is used to visually assess the presence of publication bias and asymmetry in the distribution of estimated causal effects, helping to evaluate the reliability and potential bias in the analysis; **(D)** Leave-one-out analysis, it serves as a sensitivity analysis to assess the robustness and reliability of the causal estimates by systematically excluding individual instrumental variables and evaluating their impact on the overall findings.

Supplementary Figure 7 Complementary MR analyses of hypothyroidism with BMI. **(A)** Forest plot, it provides a visual representation of the causal estimates and their corresponding confidence intervals for each IV, aiding in the assessment of individual and overall effects in the analysis; **(B)** Scatter plot, it visually displays the associations between IVs and the exposure and outcome variables, facilitating the examination of the strength and direction of these relationships and aiding in the identification of potential causal effects. The regression slops fitted by different MR methods are represented by different MR methods are represented by lines of varying colors; **(C)** Funnel plot, it is used to visually assess the presence of publication bias and asymmetry in the distribution of estimated causal effects, helping to evaluate the reliability and potential bias in the analysis; **(D)** Leave-one-out analysis, it serves as a sensitivity analysis to assess the robustness and reliability of the causal estimates by systematically excluding individual instrumental variables and evaluating their impact on the overall findings.

Supplementary Figure 8 Complementary MR analyses of hypothyroidism with WC. **(A)** Forest plot, it provides a visual representation of the causal estimates and their corresponding confidence intervals for each IV, aiding in the assessment of individual and overall effects in the analysis; **(B)** Scatter plot, it visually displays the associations between IVs and the exposure and outcome variables, facilitating the examination of the strength and direction of these relationships and aiding in the identification of potential causal effects. The regression slops fitted by different MR methods are represented by different MR methods are represented by lines of varying colors; **(C)** Funnel plot, it is used to visually assess the presence of publication bias and asymmetry in the distribution of estimated causal effects, helping to evaluate the reliability and potential bias in the analysis; **(D)** Leave-one-out analysis, it serves as a sensitivity analysis to assess the robustness and reliability of the causal estimates by systematically excluding individual instrumental variables and evaluating their impact on the overall findings.

Supplementary Figure 9 Complementary MR analyses of hypothyroidism with WCadjBMI. **(A)** Forest plot, it provides a visual representation of the causal estimates and their corresponding confidence intervals for each IV, aiding in the assessment of individual and overall effects in the analysis; **(B)** Scatter plot, it visually displays the associations between IVs and the exposure and outcome variables, facilitating the examination of the strength and direction of these relationships and aiding in the identification of potential causal effects. The regression slops fitted by different MR methods are represented by different MR methods are represented by lines of varying colors; **(C)** Funnel plot, it is used to visually assess the presence of publication bias and asymmetry in the distribution of estimated causal effects, helping to evaluate the reliability and potential bias in the analysis; **(D)** Leave-one-out analysis, it serves as a sensitivity analysis to assess the robustness and reliability of the causal estimates by systematically excluding individual instrumental variables and evaluating their impact on the overall findings.

Supplementary Figure 10 Complementary MR analyses of hypothyroidism/myxedema with BMI. **(A)** Forest plot, it provides a visual representation of the causal estimates and their corresponding confidence intervals for each IV, aiding in the assessment of individual and overall effects in the analysis; **(B)** Scatter plot, it visually displays the associations between IVs and the exposure and outcome variables, facilitating the examination of the strength and direction of these relationships and aiding in the identification of potential causal effects. The regression slops fitted by different MR methods are represented by different MR methods are represented by lines of varying colors; **(C)** Funnel plot, it is used to visually assess the presence of publication bias and asymmetry in the distribution of estimated causal effects, helping to evaluate the reliability and potential bias in the analysis; **(D)** Leave-one-out analysis, it serves as a sensitivity analysis to assess the robustness and reliability of the causal estimates by systematically excluding individual instrumental variables and evaluating their impact on the overall findings.

Supplementary Figure 11 Complementary MR analyses of hypothyroidism/myxedema with WC. **(A)** Forest plot, it provides a visual representation of the causal estimates and their corresponding confidence intervals for each IV, aiding in the assessment of individual and overall effects in the analysis; **(B)** Scatter plot, it visually displays the associations between IVs and the exposure and outcome variables, facilitating the examination of the strength and direction of these relationships and aiding in the identification of potential causal effects. The regression slops fitted by different MR methods are represented by different MR methods are represented by lines of varying colors; **(C)**. Funnel plot, it is used to visually assess the presence of publication bias and asymmetry in the distribution of estimated causal effects, helping to evaluate the reliability and potential bias in the analysis; **(D)** Leave-one-out analysis, it serves as a sensitivity analysis to assess the robustness and reliability of the causal estimates by systematically excluding individual instrumental variables and evaluating their impact on the overall findings.

Supplementary Figure 12 Complementary MR analyses of hypothyroidism/myxedema with WCadjBMI. **(A)** Forest plot, it provides a visual representation of the causal estimates and their corresponding confidence intervals for each IV, aiding in the assessment of individual and overall effects in the analysis; **(B)** Scatter plot, it visually displays the associations between IVs and the exposure and outcome variables, facilitating the examination of the strength and direction of these relationships and aiding in the identification of potential causal effects. The regression slops fitted by different MR methods are represented by different MR methods are represented by lines of varying colors; **(C)** Funnel plot, it is used to visually assess the presence of publication bias and asymmetry in the distribution of estimated causal effects, helping to evaluate the reliability and potential bias in the analysis; **(D)** Leave-one-out analysis, it serves as a sensitivity analysis to assess the robustness and reliability of the causal estimates by systematically excluding individual instrumental variables and evaluating their impact on the overall findings.

Supplementary Table 1 Characteristics of the SNPs used as IVs in the causal association of obesity-related traits with hypothyroidism.

Supplementary Table 2 Characteristics of the SNPs used as IVs in the causal association of hypothyroidism with obesity-related traits.
